# Whole Exome Sequencing Reveals Clustering of Variants of Known Vitiligo Genes in Multiplex Consanguineous Pakistani Families

**DOI:** 10.3390/genes14051118

**Published:** 2023-05-22

**Authors:** Rafaqat Ishaq, Muhammad Ilyas, Umme Habiba, Muhammad Noor ul Amin, Sadia Saeed, Ghazala Kaukab Raja, Pakeeza Arzoo Shaiq, Zubair M. Ahmed

**Affiliations:** 1 University Institute of Biochemistry and Biotechnology, Pir Mehr Ali Shah Arid Agriculture University, Rawalpindi 46300, Pakistan; rafaqatishaq@gmail.com (R.I.); noorulaminamin@yahoo.com (M.N.u.A.);; 2 Department of Otorhinolaryngology-Head and Neck Surgery, School of Medicine, University of Maryland, Baltimore, MD 20742, USA; 3 Department of Medical Laboratory Technology, Riphah International University, Malakand Campus, Malakand 23010, Pakistan; 4 Department of Clinical Molecular Biology, EpiGen, Institute of Clinical Medicine, University of Oslo, 0313 Oslo, Norway; 5 Department of Biochemistry and Molecular Biology, School of Medicine, University of Maryland, Baltimore, MD 20742, USA; 6 Department of Ophthalmology and Visual Sciences, School of Medicine, University of Maryland, Baltimore, MD 20742, USA

**Keywords:** vitiligo, non-segmental vitiligo, polygenic burden, genetic heterogeneity, allele clustering, *PTPN22*, *HERC2*, *NRROS*

## Abstract

Vitiligo is an autoimmune complex pigmentation disease characterized by non-pigmented patches on the surface of the skin that affect approximately 0.5–2% population worldwide. The exact etiology is still unknown; however, vitiligo is hypothesized to be a multifactorial and genetically heterogeneous condition. Therefore, the current study is designed to investigate the anthropometric presentation and genetic spectrum of vitiligo in fifteen consanguineous Pakistani families. The clinical evaluation of participating individuals revealed varying degrees of disease severity, with 23 years as the average age of disease onset. The majority of the affected individuals had non-segmental vitiligo (NSV). Whole exome sequencing analysis revealed clustering of rare variants of known vitiligo-associated genes. For instance, in the affected individuals of family VF-12, we identified three novel rare variants of *PTPN22* (c.1108C>A), *NRROS* (c.197C>T) and *HERC2* (c.10969G>A) genes. All three variants replaced evolutionarily conserved amino acid residues in encoded proteins, which are predicted to impact the ionic interactions in the secondary structure. Although various in silico algorithms predicted low effect sizes for these variants individually, the clustering of them in affected individuals increases the polygenic burden of risk alleles. To our knowledge, this is the first study that highlights the complex etiology of vitiligo and genetic heterogeneity in multiplex consanguineous Pakistani families.

## 1. Introduction

Vitiligo is a common skin pigmentation disorder with remarkable genetic and clinical heterogeneity. Vitiligo is characterized by the appearance of colorless patches on the epidermal surface of the skin. Discrepancies in the production and distribution of melanin in affected areas result in the onset of this socially stigmatizing and psychologically devastating disease. Vitiligo may affect one or both halves of the body. The vitiligo European task force assessment (VETFa) has broadly classified vitiligo into two main types, i.e., non-segmental vitiligo (NSV) or generalized vitiligo (GV) and Segmental Vitiligo (SV). Vitiligo is known to be progressive in most patients, which eventually results in complete loss of skin coloration, referred to as vitiligo Universalis (VU) in elderly patients. Various studies have reported that the worldwide incidence of vitiligo ranges between 0.5–2% [1,2]. However, there is no prior comprehensive study documenting the prevalence of vitiligo in the Pakistani population.

Vitiligo is a multifactorial, polygenic disorder that follows a non-Mendelian inheritance pattern with considerable incomplete penetrance due to complex genetic and environmental etiology [3]. A number of theories have been proposed to explain vitiligo pathogenesis that include genetics, autoimmune, auto-cytotoxic, and gene-environment interactions, which indicates that gene-environment interactions are predominantly involved in disease pathogenesis [4]. Multiplex vitiligo families partially support genetic hypotheses [5]. A report on the Korean population suggests a polygenic etiology of the disease [6]. The possible involvement of different sets of genes in melanin production, epistatic interactions, multistep regulation of the autoimmune system, and control of auto-toxicity has been implicated in disease pathogenesis [4]. The presence of certain environmental triggers, such as oxidative stress, ultraviolet radiation, and exposure to phenolic derivative compounds, may initiate vitiligo macule formation [7,8,9].

Advancement in genetic screening technologies has tremendously helped in the identification of rare variants associated with vitiligo. In the current study, we identified fifteen clinically diagnosed multiplex vitiligo families from the Punjab province of Pakistan. Whole exome sequencing was performed on seven of these families, which identified rare variants in vitiligo-associated genes. Our study also points towards the clinical and genetic complexity and locus heterogeneity of vitiligo in the inbred Pakistani population.

## 2. Materials and Methods

### 2.1. Family Enrollment and Clinical Classification

The proceedings of the current study were approved by committees of the Institutional Review Board (IRB) at the University of Maryland, School of Medicine, Baltimore, MD, USA (HP-00061036) and Pir Mher Ali Shah Arid Agriculture University Rawalpindi, Pakistan. The tenets of the Declaration of Helsinki were followed for the use of human subjects in the current study. Informed consent in writing was obtained from all participants. Fifteen vitiligo families were recruited from different regions of the Punjab province of Pakistan in this study.

Detailed family histories were obtained to document disease onset, affected area of skin, disease progression status, smoking/non-smoking status, chemical exposure, general lifestyle, eating habits, treatment etc. Family pedigrees were drawn based on the information received and verified by multiple participating family members.

### 2.2. Sample Collection and Isolation of DNA

From all participating individuals, 3 mL of venous blood samples were collected in an EDTA Orsin vacutainer (formerly imuMed^®^) and stored at 4 °C until further use. The genomic DNA was isolated from the blood samples of participants using the Thermo Scientific™ GeneJET genomic DNA purification kit (Catalog #: K0722). The concentration of DNA was quantified by NanoDrop^®^ ND-1000 UV-Vis Spectrophotometer (Thermo Fisher Scientific, Waltham, MA, USA), and DNA quality was assessed by 260/280 and 260/230 ratios.

### 2.3. Whole Exome Sequencing

Whole exome sequencing was performed on the probands of families VF-01, VF-02-, VF-03, VF-09, VF-10, VF-12 and VF-15. Genomic libraries were recovered for exome enrichment by using the Agilent SureSelect Human Expanded All Exon V5 kit and sequenced on an Illumina HiSeq4000 (Illumina, San Diego, CA, USA) with an average of 100× coverage [10]. Broad Institute’s Toolkit for Genome Analysis was used to analyze the sequencing data, as described previously [11,12], with the following modifications: (a) allele frequencies cut off was set to ≤0.003; (b) potential pathogenic prediction was achieved by at least 2 in silico algorithms; and (c) CADD score ≥ 6.0. Primers were designed using the online resource Primer3 (http://bioinfo.ut.ee/primer3-0.4.0/, accessed on 23 October 2018) for the variants that passed our filtration criteria; targeted DNA amplification by conventional PCR followed by Sanger sequencing was carried out for segregation studies [13].

### 2.4. Molecular Modeling

The Phyre2 (http://www.sbg.bio.ic.ac.uk/phyre2/html/page.cgi?id=index, accessed on 15 March 2019) and protein variation predictor resource HOPE (https://www3.cmbi.umcn.nl/hope/, accessed on 15 March 2019) were applied for 3D modeling of protein structures. Visualization of the mutation impact on the 3D structure and change of ionic interaction between the residues of interest was visualized by the UCSF CHIMERA tool (https://www.cgl.ucsf.edu/chimera/, accessed on 19 March 2019). MUpro (http://mupro.proteomics.ics.uci.edu/, accessed on 27 March 2023) and I-Mutant 2.0 (http://folding.biofold.org/i-mutant/i-mutant2.0.html, accessed on 27 March 2023) were used to predict the effects of these mutations on protein structure stability on the bases of changes in Gibbs free energy (G). The evolutionary conservation status of multiple amino acids across different species was performed by Clustal Omega multiple sequence alignment (https://www.ebi.ac.uk/Tools/msa/clustalo/, accessed on 10 March 2019).

## 3. Results

### 3.1. Clinical Evaluation of Vitiligo in Multiplex Pakistani Families

**Figure 1:**
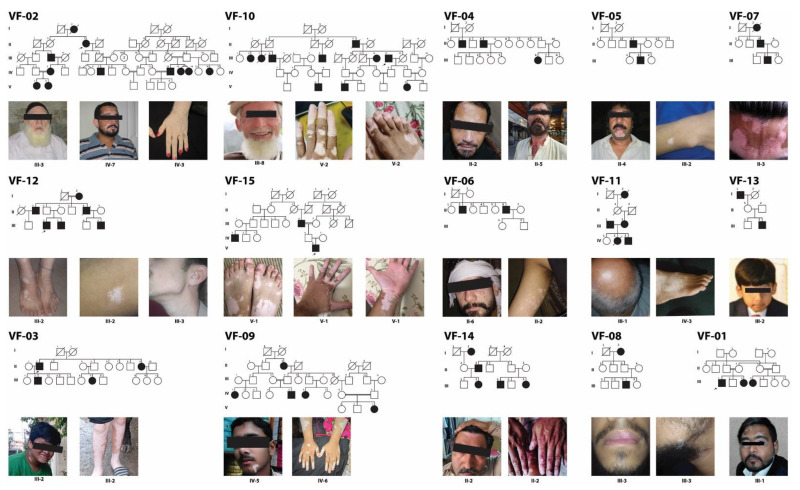
Pedigrees of vitiligo families. Black-filled and empty symbols represent affected and unaffected individuals, respectively. The double line shows only consanguineous marriages. The arrow sign indicate probands that were used for whole exome sequencing. Clinical manifestations of the vitiligo patches for representative affected members are also shown below each pedigree.

After IRB approval, a total of 15 multiplex consanguineous families segregating vitiligo (Figure 1) were enrolled in this study from five different cities (Rawalpindi, Chakwal, Mianwali, Abbottabad, Islamabad) in Pakistan (Table 1). A total of 130 individuals from these families participated in our study, including 72 males (55%) and 58 females (45%). Of these enrolled subjects, 53 (33 males and 20 females) exhibited the vitiligo phenotype, while 77 individuals had no skin problem and, thus, were used as controls in our downstream genetic studies. The age range for onset vitiligo macules was between 2 to 68 years, with an average of 23 years. Among the enrolled females, non-segmental vitiligo (NSV) was the most noticeable clinical phenotype noted in forty-eight females (91%), three females (6%) had vitiligo universalis (VU), while two females (3%) reported complete recovery from their vitiligo phenotype. Almost all affected individuals reported having no other autoimmune diseases or any other comorbidity besides vitiligo, except two affected individuals that had type 1 diabetes mellitus (Table 1).

Inter- and intra-familial variability in the areas of skin discoloration among the affected individuals was identified (Table 1). For instance, family VF-02 is an extended vitiligo family having nine affected individuals (Figure 1). Three of the affected individuals (VF02_15, _16, _17) had 90–100% of their body with vitiligo patches and thus present VU (Table 1), while the other affected individuals had much fewer patches (Table 1). Intriguingly, according to family history interviews, one affected female (VH02_07) recovered from vitiligo without any medication. In contrast, the three affected individuals of family VF-04 had relatively less progressive disease severity (Table 1).

### 3.2. Genetic Analysis of Families with Vitiligo

**Figure 2:**
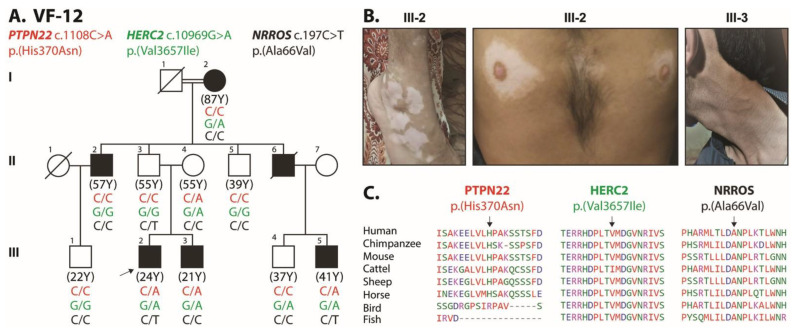
Pedigree of vitiligo family and identified causative mutation. (**A**) Polygenic pattern of disease-causing alleles segregating in multiplex Pakistani vitiligo family. Black-filled and empty symbols represent affected and unaffected individuals, respectively. Symbols filled in gray represent individuals that recovered (<5%) from vitiligo. Double line shows only consanguineous marriages. The genotypes (wild type, heterozygous) of the identified mutant alleles are also shown for each of the participating family members. Arrow sign represents the proband used for whole exome sequencing. (**B**) Vitiligo patches were observed in the affected individuals of VF-12 family. (**C**) Clustal-W multiple amino acid sequence alignments of orthologous proteins show evolutionarily conserved mutated residues across different species.

To decipher the genetic risk factors contributing to the vitiligo phenotype in the enrolled families, we performed whole exome sequencing on index patients from seven families along with ethnically matched normal control samples. After quality control, we used in silico panel to filter variants in the known vitiligo-associated genes [14]. Next, we selected variants of the genes that had (a) an allele frequency of ≤0.001; (b) a CADD score of ≥7.0; and (c) predicted damage by at least one in silico algorithm. Table 2 lists all variants of known vitiligo genes that passed these criteria. Although various in silico algorithms predicted low effect size for these variants individually, the clustering of these variants in affected individuals increases the polygenic burden of risk alleles (Table 2); we found potentially pathogenic variants of three known vitiligo-associated genes, *PTPN22*, *HERC2* and *NRROS*, in a varying combination of zygosity among affected individuals of family VF-12 (Figure 2). Proband III-2, having the most severe phenotype, was found heterozygous for the rare missense variants (Figure 2) of *PTPN22* (c.1108C>A; p.(His370Asn)), *HERC2* (c.10969G>A; p. (Val3657Ile)), and *NRROS* (c.197C>T; p.(Ala66Val)). Different family members inherited different combinations of these potentially pathogenic variants, and hence disease severity and allelic burden vary among these individuals (Figure 2 and Table 1). Variants found in the *PTPN22*, *HERC2* and *NRROS* genes have been previously known for their association with NSV in different ethnicities around the world [15,16,17,18].

**Figure 3:**
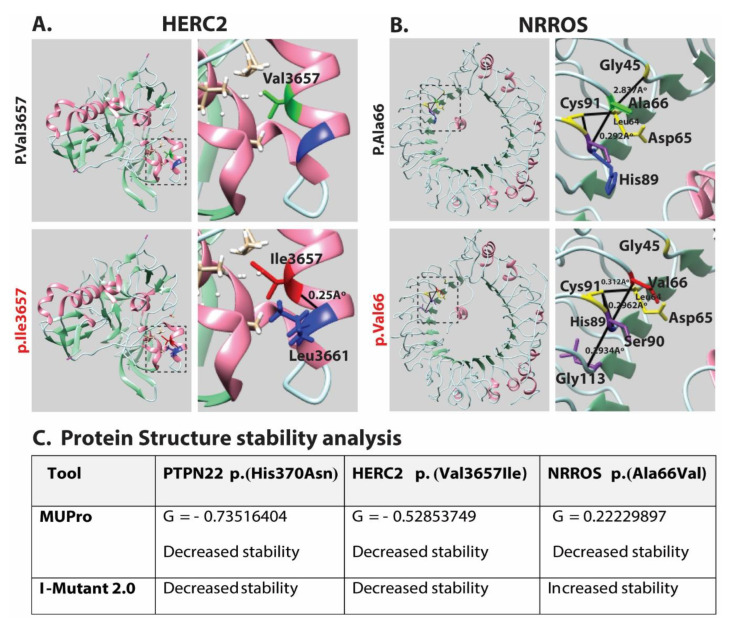
Three-dimensional protein structural modeling encoded from variants identified in the current study. Structures of both wild type (shown in green) and mutated residues (shown in red) are highlighted. Solid black lines represent the interactions with neighboring amino acids. Stick representation in green shows wild type residue, while red color shows mutated amino acid. Helices are shown in magenta, sheets in light green and loops in cyan color. (**A**) Missense variant in HERC2 p.(Val3657Ile), wild type residue is relatively smaller in size than the mutant. Mutant residue p.(Ile 3657), shown in red, forms H-bonds with neighboring p.(Leu 3661), shown in blue, and bond length is 0.2561 Å, which may have an impact on protein folding and functionality. (**B**) Similarly, for missense variant of NRROS p.(Ala66Val), wild type residue p.(Ala66), shown in green, forms H-bonds with p.(His89), shown in blue. Neighboring residue, shown in yellow with solid black line, represents H-bonds in the wild type. Mutant model represents different sets of interactions in the adjacent region of mutation. p.(Val66), shown in red, forms H-bonds with p.(His89), shown in blue, with different bond lengths causing loss of H-bonding between p.(Gly45) and p.(Asp65), shown in yellow, in wild type. Formation of new intra residual H-bonding between p.(Ser90) and p.(Gly113) is shown in violet color. (**C**) The underlying mutations negatively impacted protein structures, and the encoded proteins were predicted to become unstable by MUpro and I-mutant.

Amino acid conservation studies have shown that the variants identified in *PTPN22*, *HERC2* and *NRROS* genes are present in the evolutionarily conserved regions of the encoded proteins (Figure 2C). Next, we performed molecular modeling analysis using the Phyre2 online resource for retrieving structural information based on protein homology present in the protein database. Using this approach, we were able to model the two missense variants found in the HERC2 and NROSS proteins (Figure 3). The lack of available crystalline structural information for PTPN22 restrained further analysis from predicting the effect of the mutation on protein structure and function. A different pattern of interresidual bonding interaction between wild type and mutant protein was observed for the p.Val 3657Ile variant of HERC2 (Figure 3A). The modeling data predicted that p.Val3657Ile substitution might impact protein folding due to the formation of an additional H-bond between p.Ile3657 and p.Leu3661 (Figure 3A). Similarly, the p.Ala66Val variant of NRROS is predicted to greatly hamper the microscopic interaction in the surrounding area of 10 Å by differential H-bonding patterns (Figure 3B). Surrounding the p.Ala66 residue in WT NRROS, there are four prominent hydrogen bonds: between p.Gly45 and p.Asp65 (2.837 Å); p.Cys91 and Leu64 (0.3129 Å); p.Cys91 and p.His89 (0.374 Å); and p.Ala66 and p.His89 (2.921 Å; Figure 3B). In contrast, the p.Ala66Val substitution exhibits a different set of H-bonding capabilities, resulting in the loss of H-bonds between p.Gly45 and p.Asp65 and the formation of two new H-bond bonds between p.Val66 and p.His89 (2.962 Å), and between p.Gly113 and p.Ser90 (2.293 Å; Figure 3B). This new pattern of interaction may induce torsion, leading to improper protein folding and properties, which may have an effect on function. A noticeable decrease in Gibbs free energy was recorded in PTPN22 (−0.73516404), HERC2 (−0.52853749) and NRROS (−0.22229897) by MUpro, indicating a decrease in protein 3D structure stability, as corroborated by the I-Mutant prediction program. Overall, the three identified variants have been anticipated to have deleterious effects on protein structures, rendering the proteins unstable (Figure 3C).

**Figure 4:**
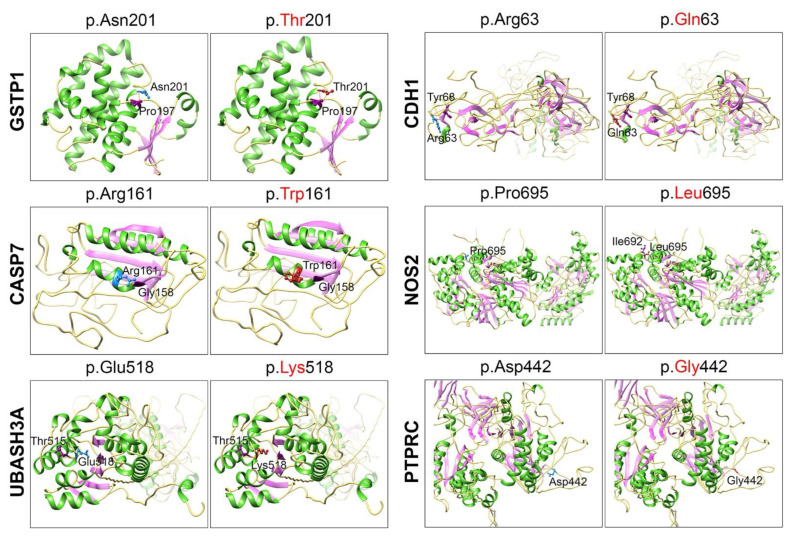
WT residue is shown in dodger blue color, mutant in firebrick (red) and hydrogen-bond (orange color line) associated amino acids in dark magenta. Amino acid atoms are shown in ball and stick forms. Secondary structure: Helix: Green strands: pink-purple and coils: golden-yellow in color.

Using this in silico approach, we also modeled six missense variants found in the affected individuals of our vitiligo families (Table 2) and predicted their potential pathogenic impact on the encoded proteins (Figure 4). These include p.Asn201Thr (GSTP1), p.Arg63Gln (CDH1), p.Arg161Trp (CASP7), p.Pro695Leu (NOS2), p.Glu518Lys (UBASH3A), and p.Asp442Gly (PTPRC) variants (Figure 4). The p.Asn201Thr variant of GSTP1 is located within the GST C-terminal domain, and due to different sizes and ionic properties, it might impact the folding of the domain (Figure 4). A similar impact was predicted for the p.Arg63Gln variant of CDH1, p.Arg161Trp of CASP7, and p.Asp442Gly of PTPRC (Figure 4). The p.Pro695Leu of NOS2 is predicted to form a new H-bond with p.Ile692 (Figure 4). Moreover, the WT Proline residue is known to be very rigid and therefore induce a special backbone conformation; therefore, substitution with Leucine would affect this conformation. Finally, the p.Glu518Lys variant of UBASH3A is predicted to alter the salt bridge with Arginine at position 406 and Arginine at position 485 (Figure 4), and thus it ultimately affects protein folding and function. Overall, the above missense variants are predicted to have an impact on protein folding and secondary structure, which might lead to impaired function.

## 4. Discussion

Vitiligo is a disease of complex etiology, whereby the involvement of genetic contributors in disease pathogenesis has been emphasized greatly since the advent of modern genetic technologies. Previously, several genetic association studies have identified risk alleles responsible for various types of vitiligo in different ethnicities [17,19,20,21,22,23,24]. In the present study, we reported clinical and genetic findings in fifteen highly inbred consanguineous Pakistani families. Our study highlights the genetic heterogeneity of the disease in terms of risk allele clustering as well as associated risk allele burden in affected individuals. We report the identification of several rare variants of known vitiligo-associated genes, including three novel missense variants in *PTPN22* (c.1108C>A; p.(His370Asn)), *NRROS* (c.197C>T; p.(Ala66Val)) and *HERC2* (c.10969G>A; p. (Val3657Ile)) in family VF-12. In silico analysis of the risk variants for pathogenicity determination predicted a low to moderate pathogenic impact of all the identified variants; however, clustering of mutated alleles in an individual collectively contributed to the onset of vitiligo.

Autoimmune-mediated melanocyte destruction is a common phenomenon in vitiligo pathogenesis that involves intrinsic defects within melanocytes and an autoimmune system that targets these cells [25]. *PTPN22*, which encrypts genetic information for lymphoid-specific intracellular phosphatase, is the main inhibitor of T-cell activation [19]. Many autoimmune diseases, including type 1 diabetes, arthritis, systemic lupus and vitiligo, have also been previously associated with *PTPN22* variants [4]. NSV was found to be strongly associated with *PTPN22* (c.1858C>T; p. (Arg620Trp)) in the cohorts from the United Kingdom, Gujarat, India and Romania in three different case-control studies [15,26]. Similarly, *HERC2*, which encodes for the E3 ubiquitin-protein, provides protection against ionizing radiation and DNA damage related to radiation. Previous studies have shown that an elevated risk of vitiligo is associated with *HERC2* variations, which have a significant impact on determining skin and iris color besides the *OCA2*. Previous studies on Caucasian and Chinese Han cohorts also reported a significant association between the T allele of *rs6583331* SNP of *NRROS* and vitiligo [17]. However, in our cohort, a novel vitiligo-associated variant of *NRROS* was identified. Our outcomes are in agreement with previous findings, thus indicating an increased risk for intra-familial NSV development linked to *NRROS.*

Besides the above-mentioned genes, as shown in Table 2, the majority of the variants identified in our cohort are present in proteins that function in innate immune responses, inflammation reaction, control of cell apoptosis, anti-oxidative damage, and have a role in detoxification reactions. For instance, IFIH1 is an interferon-induced helicase C domain-containing protein, an innate immune receptor that acts as a cytoplasmic sensor of viral nucleic acids and plays a major role in sensing viral infection and in the activation of a cascade of antiviral responses, including the induction of type I interferons and pro-inflammatory cytokines [25]. The IL1RAPL1-Interleukin-1 receptor accessory protein-like 1 regulates secretion and presynaptic differentiation through inhibition of the activity of the N-type voltage-gated calcium channel and is also involved in the activation of the MAP kinase JNK. TLR4 is a transmembrane protein, a member of the Toll-like receptor family, which belongs to the pattern recognition receptor (PRR) family. TLR4 participates in intracellular signaling NF-κB and inflammatory cytokine production, which is responsible for activating the innate immune system. *CASP7* encodes a member of the Cysteine-Aspartic acid protease (caspase) family and plays a central role in the execution phase of cell apoptosis and survival of melanocytes [4]. Similarly, the BCL2L12 protein family and their interactors include inhibitors and inducers of cell death, regulate and mediate the process by which mitochondria contribute to cell death known as the intrinsic apoptosis pathway, and thus regulate the survival of pigmented cells.

In conclusion, the convergence theory of vitiligo development bridges all previously known etiologies and describes vitiligo as a multivariate, complex, and multifactorial disorder [9]. Vitiligo is not caused merely by the presence of an underlying genetic variation or only by exposure to some specific external factor such as stimuli or defective melanocytes and an internal autoimmune condition but is a result of an intricate combination of all these etiological contributors. Therefore, it is important to acknowledge that the underlying molecular pathways affected in one patient do not always lead to the same level of pathogenicity in other individuals. Such attributes of vitiligo define that identification of individual-specific pathways is significant for the targeted development of more effective therapeutics [26]. Polygenic Risk Score (PRS) estimation is a trending approach for the genetic description of complex disorders, including vitiligo. However, there always remains quite a fair chance for the involvement of additional genes or novel variants in the known vitiligo-associated genes. In this regard, it has been recently described that the phenomenon of clustering of risk alleles in an inbred family increases polygenic risk burden [5], and our study further supports this notion.

## Figures and Tables

**Table 1:** Clinical phenotype of Vitiligo families.

Family ID	Family Ethnicity	Individual ID	Age (Year)	Age of Onset	Gender	Phenotype	Doctoral Description of the Disease	Affected Area of Skin (%)	Comorbidity
**VF-01**	Rajput	VF01_01	28	18	Male	Affected	NSV, Generalized vitiligo	20–30%	Nil
		VF01_02	26	21	Female	Affected	NSV, Generalized Vitiligo	20–30%	Nil
		VF01_04	17	14	Female	Affected	NSV, Generalized vitiligo	10–20%	Nil
**VF-02**	Mughal	VF02_03	28	20	Female	Affected	NSV, Generalized vitiligo	10–20%	Nil
		VF02_06	18	18	Female	Affected	NSV, Generalized Vitiligo	Less than 5%	Nil
		VF02_07	42	39	Male	Recovered	Generalized Vitiligo (NSV*)	N/A	Nil
		VF02_11	32	17	Male	Affected	NSV, Generalized vitiligo	30–40%	Nil
		VF02_15	80	14	Female	Affected	Vitiligo Universalis	90–100%	Diabetes
		VF02_16	79	19	Male	Affected	NSV, Generalized vitiligo	90–100%	Nil
		VF02_17	56	18	Female	Affected	NSV, Generalized vitiligo	90–100%	Nil
		VF02_18	23	11	Female	Affected	NSV, Generalized vitiligo	10–20%	Nil
		VF02_19	20	10	Female	Recovered	Vitiligo recovered (NSV*)	N/A	Nil
**VF-03**	Awan	VF03_01	46	24	Male	Affected	NSV, Generalized vitiligo	30–40%	Nil
		VF03_07	7	04	Male	Affected	NSV, Acrofacial vitiligo	5–10%	Nil
		VF03_08	30	27	Female	Affected	NSV, Acrofacial vitiligo	Less than 5%	Nil
**VF-04**	Sardar	VF04_01	30	24	Male	Affected	NSV, Generalized vitiligo	20–30%	Nil
		VF04_02	38	31	Male	Affected	NSV, Generalized vitiligo	Less than 5%	Nil
		VF04_03	13	7	Female	Affected	NSV, Generalized vitiligo	Less than 5%	Nil
**VF-05**	Mughal	VF05_01	47	33	Male	Affected	NSV, Generalized vitiligo	20–30%	Nil
		VF05_04	22	21	Male	Affected	Vitiligo started in neck region	Less than 5%	Nil
**VF-06**	Rajput	VF06_01	31	11	Male	Affected	NSV, Generalized vitiligo	70–80%	Nil
		VF06_02	25	13	Male	Affected	NSV, Generalized vitiligo	40–50%	Nil
**VF-07**	Niazi	VF07_01	55	23	Male	Affected	NSV, Generalized vitiligo	40–50%	Nil
		VF07_03	21	19	Male	Affected	NSV, Generalized vitiligo	5–10%	Nil
**VF-08**	Shiekh	VF08_01	69	19	Female	Affected	NSV, Generalized vitiligo	20–30%	Nil
		VF08_04	21	17	Male	Affected	NSV, Generalized vitiligo	5–10%	Nil
**VF-09**	Awan	VF09_01	64	59	Female	Affected	NSV, Generalized vitiligo	20–30%	Nil
		VF09_05	17	15	Male	Affected	NSV, Generalized vitiligo	Less than 5%	Nil
		VF09_07	19	15	Female	Affected	NSV, Generalized vitiligo	Less than 5%	Nil
		VF09_10	9	2	Female	Affected	NSV, Generalized vitiligo	20–30%	Nil
**VF-10**	Malik	VF10_01	79	33	Male	Affected	NSV, Generalized vitiligo	60–70%	Nil
		VF10_04	83	42	Male	Affected	NSV, Generalized vitiligo	70–80%	Nil
		VF10_07	80	40	Female	Affected	NSV, Generalized vitiligo	40–50%	Nil
		VF10_10	35	27	Male	Affected	NSV, Generalized vitiligo	40–50%	Nil
		VF10_11	51	33	Male	Affected	NSV, Generalized vitiligo	50–60%	Nil
		VF10_14	12	7	Male	Affected	NSV, Generalized vitiligo	5–10%	Nil
		VF10_16	28	17	Male	Affected	NSV, Generalized vitiligo	40–50%	Nil
**VF-11**	Qazi	VF11_01	56	11	Female	Affected	Vitiligo Universalis	90–100%	Nil
		VF11_02	58	53	Male	Affected	Vitiligo just started at the skull	Less than 1%	Nil
		VF11_03	19	11	Male	Affected	NSV, Generalized vitiligo	5–10%	Nil
**VF-12**	Malik	VF12_01	57	43	Male	Affected	NSV, Generalized vitiligo	30–40%	Diabetes
		VF12_05	26	15	Male	Affected	NSV, Generalized vitiligo	30–40%	Nil
		VF12_06	23	22	Male	Affected	NSV, Generalized vitiligo	Less than 5%	Nil
		VF12_07	35	24	Male	Affected	NSV, Generalized vitiligo	20–30%	Nil
		VF12_09	79	68	Female	Affected	NSV, Generalized vitiligo	5–10%	Nil
**VF-13**	Rajput	VF13_01	74	66	Male	Affected	NSV, Generalized vitiligo	30–40%	Nil
		VF13_04	13	9	Male	Affected	NSV, Generalized vitiligo	5–10%	Nil
**VF-14**	Chaudary	VF14_01	67	31	Female	Affected	NSV, Generalized vitiligo	15–20%	Nil
		VF14_03	47	38	Male	Affected	NSV, Generalized vitiligo	30–40%	Nil
		VF14_04	15	9	Female	Affected	NSV, Generalized vitiligo	10–20%	Nil
**VF-15**	Rajput	VF15_01	77	19	Male	Affected	Vitiligo Universalis	90–100%	Nil
		VF15_04	21	14	Male	Affected	NSV, Generalized vitiligo	20–30%	Nil
		VF15_07	21	13	Male	Affected	NSV, Generalized vitiligo	30–40%	Nil

NSV: Non segmental vitiligo; NSV*: Non-segmental vitiligo (Recovered); N/A: Not applicable.

**Table 2:** Genetic analysis of index patients for known vitiligo gene variants.

Family	Chr	Gene	Ref	Alt	c.DNA Change	Protein Change	SNP ID	rs Number	ExAC	CADD	SIFT	Polyphen	Mutation Taster	Mutation Assessor	Genotyping of Proband
**VF-01**	2	*IFIH1*	T	C	c.A1046G	p.K349R	NM_022168	rs72650664	0.0035	14.59	T	B	D	N	Het
	11	*GSTP1*	A	C	c.A601C	p.N201H	NM_000852	rs145957405	-	12.59	D	P	D	M	Het
	11	*GSTP1*	A	C	c.A602C	p.N201T	NM_000852	rs376074280	0.00001677	7.554	T	B	D	M	Het
	12	*ATXN2*	TGC	-	c.534_536del	p.178_179del	NM_002973	rs193922927	0	-	-	-	-	-	Het
	15	*HERC2*	CT	-	c.5780_5781de	p.K1927fs	NM_004667	-	-	-	-	-	-	-	Het
	16	*RPGRIP1L*	T	A	c.A278T	p.E93V	NM_015272	rs765876839	0.00000825	18.56	T	P	D	L	Het
	X	*IL1RAPL1*	TG	-	c.63_64del	p.V21fs	NM_014271	rs147274241	.	.	.	.	.	.	Het
**VF-02**	19	*BCL2L12*	G	A	c.G542A	p.R181Q	NM_138639	rs762286281	0.0003	19.56	T	D	D	L	Het
**VF-03**	8	*TG*	C	T	c.C3902T	p.P1301L	NM_003235	rs549184203	0.0002	21.1	D	D	D	M	Het
	11	*GSTP1*	A	G	c.A1G	p.M1V	NM_000852	rs1448870282	-	10.05	D	B	-	-	Het
**VF-09**	3	*GPX1*	C	A	c.G442T	p.A148S	NM_001329455	rs6446261	-	6.871	T	B	D	L	Het
	3	*GPX1*	GCC	-	c.33_35del	p.11_12del	NM_001329455	rs17838762	-	-	-	-	-	-	Het
	9	*TLR4*	A	G	c.A1084G	p.K362E	NM_003266	rs539153708	0.0002	12.84	T	P	N	N	Het
	10	*CASP7*	C	T	c.C481T	p.R161W	NM_033340	rs185649982	0	0	T	B	N	-	Het
	21	*UBASH3A*	G	A	c.G1666A	p.E556K	NM_018961	rs116752327	0.0001	15.6	D	D	D	M	Het
**VF-10**	1	*PTPRC*	T	A	c.T566A	p.I189N	NM_002838	rs201715157	0.0013	7.444	D	B	N	L	Het
	2	*FARP2*	A	G	c.A134G	p.H45R	NM_014808	rs150312458	0.0034	8.045	T	B	N	N	Het
	8	*TG*	G	A	c.G2963A	p.R988H	NM_003235	rs16893332	0.0062	11.02	T	B	P	N	Het
	14	*STRN3*	G	T	c.C53A	p.P18H	NM_001083893	rs1036923688	.	15.33	D	B	D	N	Het
	16	*CDH1*	G	A	c.G188A	p.R63Q	NM_004360	rs587780117	0.00005765	12.03	T	B	N	M	Het
	17	*NOS2*	G	A	c.C2084T	p.P695L	NM_000625	rs767807366	0.00004954	19.85	T	P	D	M	Het
**VF-12**	1	*PTPN22*	G	T	c.C1108A	p.H370N	NM_015967	rs72650671	0.0023	6.752	T	P	N	M	Het
	10	*ACTA2*	C	T	-	Splicing	-	rs112687898	-	26	-	-	D	-	Het
	15	*HERC2*	C	T	c.G10969A	p.V3657I	NM_004667	rs139953376	0.0025	3.685	T	B	N	N	Het
	20	*RALY*	G	A	c.G694A	p.G232S	NM_016732	rs779745009	0.0085	7.476	T	B	N	N	Het
**VF-15**	1	*PTPRC*	A	G	c.A1808G	p.D603G	NM_002838	rs754699279	0.00004967	12.77	T	P	D	L	Het
	3	*TGFBR2*	G	A	c.G1283A	p.R428H	NM_001024847	rs143095746	0.0004	17.14	D	B	D	L	Het
	3	*GPX1*	GCC	-	c.33_35del	p.11_12del	NM_001329455	rs17838762	-	-	-	-	-	-	Het

CADD: Combined Annotation Dependent Depletion, https://cadd.gs.washington.edu/, accessed on 4 February 2019; ExAC: Exome Aggregation Consortium, http://exac.broadinstitute.org/, accessed on 4 February 2019; SIFT: Sorting intolerant from tolerant, https://sift.bii.a-star.edu.sg/, accessed on 4 February 2019; Mutation Taster: Mutation impact testing, http://www.mutationtaster.org/, accessed on 4 February 2019.

## Data Availability

The data presented in this study are available on request from Corresponding authors. The data is not publically available due to confidentiality of patient’s information.
